# An Evaluation of PROMIS Health Domains in Sarcoma Patients Compared to the United States Population

**DOI:** 10.1155/2019/9725976

**Published:** 2019-01-16

**Authors:** Benjamin Wilke, Anna Cooper, Mark Scarborough, C. Parker Gibbs, Andre Spiguel

**Affiliations:** Division of Orthopaedic Oncology, University of Florida, Gainesville, FL, USA

## Abstract

**Background:**

The Patient Reported Outcomes Measurement Information System (PROMIS) is a patient-directed system that allows comparisons across medical conditions. With this tool, comparisons can now be made between rare conditions, such as sarcomas, and more common ailments, of the United States general population. This allows comparisons between rare conditions, such as sarcomas, to more common ailments, or even the United States (US) general population.

**Objectives:**

Our purpose was to use PROMIS to compare outcomes in patients that had undergone resection of a nonmetastatic sarcoma to the US population.

**Methods:**

One hundred thirty-eight patients were included in the analysis. These patients were divided into early (<2 years) and late follow-up (>2 years).

**Results:**

We evaluated results from seven health domains and found significantly lower scores in the physical function and depression domains. These differences were present in both the early and late cohorts when compared to the US population.

**Conclusion:**

While physical function was found to be worse in the sarcoma cohorts, we observed significantly improved levels of depression in these patients when compared to the US population. This finding was maintained over time and is an important reminder that a patient's goals and desires change following a cancer diagnosis and must be taken into consideration when planning treatment and determining a successful outcome.

## 1. Introduction

Healthcare providers historically have focused on physician-directed scoring systems and survival statistics to determine “success” in treating various conditions, cancer included. The error in this strategy comes in failing to realize that how a physician and patient define success may be widely different. The Patient Reported Outcomes Measurement Information System (PROMIS) is a new patient-reported scoring system that was developed under the National Institutes of Health (NIH) and is being widely adopted [1, 2]. It has the advantage over previous outcome measurement systems in that it is completely patient-reported and has the ability to convert raw scores to *T*-scores in order to compare these values across medical conditions. In this way, we can compare our sarcoma patients to those with more common ailments or even the general United States (US) population. This system will afford us a better understanding of what constitutes a successful outcome from the patient perspective and allow us to provide care more in line with their goals and desires.

We utilized the PROMIS to evaluate health domains of patients who had a diagnosis of nonmetastatic sarcoma and had previously undergone surgical resection. We aimed to compare these values to those of the US population to identify differences. Additionally, we separated the sarcoma cohort into early (<2 years) and late (>2 years) groups based on the time from their last surgical procedure to determine if the differences that were found were dependent on the proximity from the surgical intervention.

## 2. Patients and Methods

**Figure 1:**
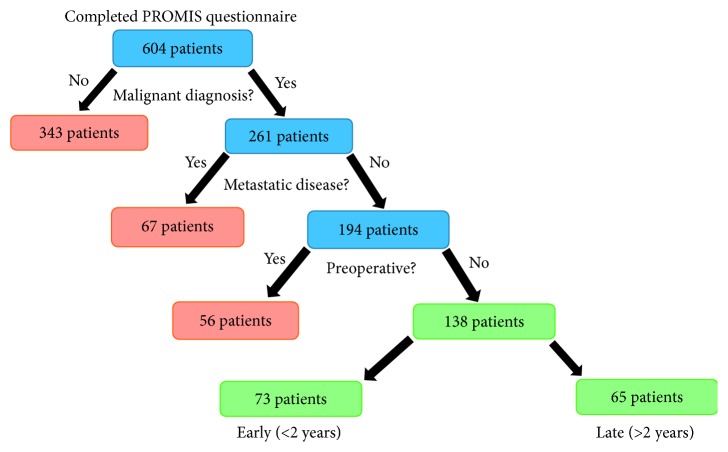
Flow chart of inclusion criteria.

PROMIS measures were obtained on all clinic patients beginning September 1, 2016. After Institutional Board Review (IRB) approval, we queried the data from September 1 through December 31, 2016. Six hundred four patients completed the PROMIS questionnaire. We excluded all patients with benign disease, those with metastatic disease, and those who had yet to undergo an operation. This left 138 patients in the final cohort with a diagnosis of nonmetastatic sarcoma who had already undergone a resection. These patients were then further divided into an early group and a late group as defined by less than or more than two years from the last surgical date (Figure 1).

Demographic data, pathologic diagnoses, and operative reports were obtained from chart review. The PROMIS 43 profile which collects short-form data for seven health domains was used. These domains include physical function, anxiety, depression, fatigue, sleep disturbance, ability to participate (in social activities), and pain interference. If patients completed more than one evaluation during the study period, then the latest questionnaire was used. The raw scores were converted to *T*-scores in order to allow comparisons with the United States general population. In the PROMIS system, the US reference population is normalized to a *T*-score of 50 with a standard deviation of 10. If a patient has a *T*-score below 50, they have less of the tested domain. Conversely, if a patient's score is above 50, then the opposite is true.

There were 77 males (56%) and 61 females (44%) included in the analysis. The average age was 57 years (range 18–94). There were 27 (20%) patients who had a sarcoma in the upper extremity and 111 (80%) with a sarcoma in the lower extremity. Seventeen (12%) of the patients had a prior inadvertent excision prior to definitive surgery at our institution. The average time between the last surgery and the survey was 11 months in the early cohort and 72 months in the late cohort. One hundred fourteen patients (83%) underwent a limb salvage procedure. Sixty-six patients (47%) received radiation therapy, and 37 patients (27%) were given chemotherapy. Radiation therapy was given as neoadjuvant treatment in most circumstances, with adjuvant treatment reserved for close margins at the time of resection or for patients that underwent re-excision of a previous inadvertent excision. Chemotherapy was given for a diagnosis of Ewing's sarcoma, osteosarcoma, and synovial sarcoma. It was also provided in a limited setting to young patients with nonmetastatic soft-tissue sarcomas at the discretion of the treating medical oncologist. Patient demographics are listed in Table 1.

## 3. Statistical Analysis

PROMIS survey results were calculated according to the PROMIS scoring manuals. We converted raw scores to *T*-scores. If a patient did not complete all the questions for a given domain, then we interpolated the result using a weighted mean formula as directed by the scoring manual guidelines.

Demographics were compared by the chi-squared test or ANOVA as indicated for group differences. For multilevel ANOVA, interactions between independent variables were analyzed. We used the one-sample Wilcoxon ranked signed test with a significance of 0.05 to determine whether *T*-scores differed from the US population mean. All analyses were performed in SPSS (IBM SPSS Statistics V24.0) and two-sided *P* values of 0.05 were considered significant.

## 4. Results

One hundred thirty-eight patients were included in the study. These were divided into early and late cohorts as defined by their last surgical procedure. The early cohort was within 2 years of their latest surgery, and the late cohort was >2 years from their surgical procedure.

In the early surgical cohort, there were 51 patients (70%) who underwent resection of a soft-tissue sarcoma and 22 (30%) who underwent resection of a primary bone tumor. Sixteen patients (22%) underwent amputation. These included three ray resections, four above-knee amputations, three hip disarticulations, four below-knee amputations, and two hemipelvectomies.

In the late cohort, there were 38 (58%) soft-tissue sarcomas and 27 (42%) primary bone tumors. Eight patients (12%) underwent an amputation. Amputations included three above-knee amputations, two below-knee amputations, one ray resection, one hip disarticulation, and one hemipelvectomy.

When comparing cohorts, we found no difference in the gender, location of the tumor, average age of the patient, or history of inadvertent excision. We also found no significant difference in the average pain scores between these groups (Table 1).

**Figure 2:**
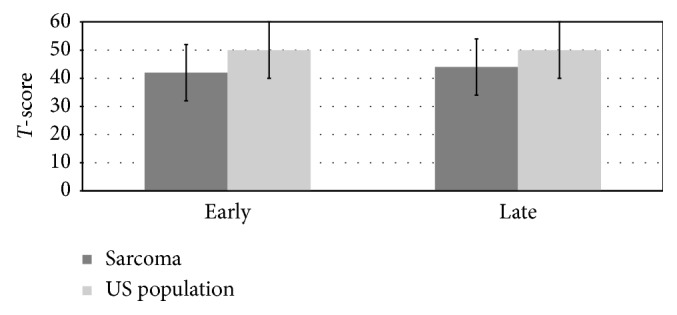
Physical function PROMIS values.

**Figure 3:**
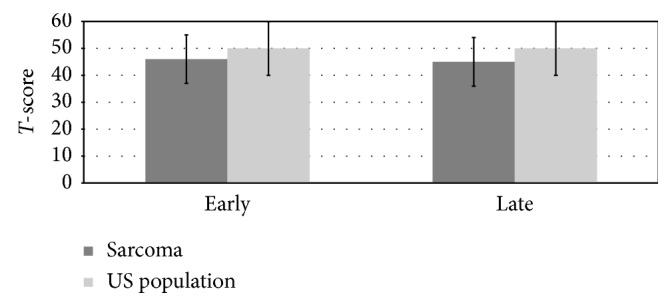
Depression PROMIS values.

We found several significant differences in the PROMIS health domains between the early and late cohorts and the US general population (Table 2). The physical function score in the early cohort was significantly reduced when compared to the US general population. This score remained significantly lower than the US population in the late cohort as well. This is represented graphically in Figure 2. Additionally, we found a significant reduction in the depression *T*-scores in both the early and late cohorts when compared to the US general population (Figure 3). Notably, 47% of the early cohort and 59% of the late cohort scored the lowest (best) score for depressive symptoms.

We were unable to find a significant difference in the anxiety, fatigue, sleep disturbance, ability to participate, or pain interference scores in either the early or late sarcoma cohorts when compared to the US general population.

## 5. Discussion

Previous reports have focused on survival statistics and physician-directed scoring tools, such as the Musculoskeletal Tumor Society (MSTS) scoring system to evaluate success following surgical interventions [3–10]. The error in this approach is that what we as physicians assume to be a successful outcome may not be in line with the patient's expectations, goals, or desires. A benefit of a purely patient-derived scoring tool, such as PROMIS, is that we obtain a much clearer picture of how the patient perceives their outcome. With the added ability to standardize these values for comparison across medical conditions or even the US general population, PROMIS becomes a powerful tool. In spite of the advantages of the PROMIS questionnaire compared to previous systems, it has not routinely been utilized in oncologic research [11–13].

In this study, we utilized the PROMIS to evaluate health domains of patients with a diagnosis of nonmetastatic sarcoma who had undergone a surgical resection and were potentially cured of their disease. We compared these patients to the United States general population to determine if there were differences in quality-of-life metrics. Additionally, we divided patients into two cohorts, those less than 2 years from their surgical resection and those greater than 2 years, to determine if any of the differences were dependent on time from the surgical intervention.

We found significant differences in two of the seven health domains. These included physical function and depression. In the early cohort, we observed an average physical function score of 42. This was significantly lower than the US general population's average of 50 and indicates that these patients were identified as having lower overall physical function ability and increased difficulty with activities of daily living. Similarly, the late cohort also demonstrated a lower average score (44) when compared to the US general population. These results are not surprising as the cohorts combined patients who underwent limb salvage and amputative procedures. Previous research has demonstrated that patients who underwent large resections and amputative procedures had lower functional scores [14, 15].

In addition to physical function, we also noted significant differences in the depression health domain. Again, we found a significantly lower average value (46) in the early cohort compared to the US general population. This value remained low in the late cohort as well, whereas in the physical function domain, a lower score is undesirable and a lower score in the depression domain suggests that patients perceive themselves as suffering less from depression than the US general population.

While it may seem counterintuitive that patients with cancer demonstrate improved depression levels when compared to the United States general population, several previous studies have reported this exact finding [13, 16–18]. In a study by Groenvold et al. that compared breast cancer patients to the Danish general population, they found lower anxiety and depression in the breast cancer cohort. Similarly, Gradl et al. compared rotationplasty patients to a healthy German sample cohort and again noted increased social functioning and mental health scores in the rotationplasty patients compared to the general population [19].

Similar research has been performed on subjects who perceived themselves to have an uncertain future. This research, performed by Carstensen et al., noted a difference in goals and desires in elderly and young subjects, with the elderly choosing more emotionally-meaningful experiences. Interestingly, they noticed that following periods of crises, such as following the September 11 attacks or the SARS outbreak, the young subjects align with the elderly in choosing the emotionally meaningful experiences. The researchers argue that an individual's goals and desires will be determined by the amount of perceived time they have left or if their future becomes uncertain, irrespective of age [20–23]. This research may help explain the differences in depression observed in our study between the sarcoma patients and the United States general population as the sarcoma patients may perceive their future to be more uncertain following their diagnosis of cancer. Future prospective studies will help determine how the depression score changes over time.

We have several limitations in this study. Our numbers are limited due to the rarity of sarcomas. This research is from a single institution, and results may not be generalizable. Our groups were heterogeneous with respect to tumor types and locations as well as treatment. With more homogenous and larger groups, we may find more differences in the PROMIS values. Additionally, we observed a flooring effect with many of the patients in the cohorts reporting the lowest possible depression scores. With a more sensitive test, we may find an even larger difference in the depression scores between the cohorts and the US population.

## 6. Conclusion

Significant differences were found in the PROMIS physical function and depression health domains when comparing patients with nonmetastatic sarcoma to the United State general population. While physical function was found to be worse in the sarcoma cohorts, we also observed significantly improved levels of depression in these patients when compared to the US population. This finding was maintained over time and is an important reminder that a patient's goals and desires change following a cancer diagnosis and must be taken into consideration when planning treatment and determining a successful outcome.

## Figures and Tables

**Table 1:** Patient demographics.

	Early (*N*=73)	Late (*N*=65)	Total (*N*=138)	*P* value
Sex				
Female	33	28	61	0.803
Male	40	37	77	
Upper extremity				
Yes	17	10	27	0.246
No	56	55	111	
Prior resection				
Yes	11	6	17	0.301
No	62	59	121	
Age (mean ± SD in months)	59.8 (±17.6)	54.7 (±19.4)		0.112
Time from surgery to survey (mean ± SD in months)	11 (±7)	72 (±58)		<0.001
Limb salvage				
Yes	57	57	114	0.139
No	16	8	24	
Average pain score (mean ± SD)	3.63	4.12		0.28

**Table 2:** PROMIS values.

Early vs. late follow-up		Mean	SD	US population	SD	*P* value
Physical function *T*-score	Early	42	11	50	10	**<0.001**
	Late	44	10			**<0.001**
Anxiety *T*-score	Early	50	9	50	10	0.567
	Late	49	10			0.309
Depression *T*-score	Early	46	9	50	10	**0.001**
	Late	45	9			**<0.001**
Fatigue *T*-score	Early	48	11	50	10	0.062
	Late	48	12			0.182
Sleep disturbance *T*-score	Early	48	9	50	10	0.082
	Late	48	11			0.169
Ability to participate *T*-score	Early	48	12	50	10	0.208
	Late	51	11			0.245
Pain interference *T*-score	Early	53	11	50	10	0.085
	Late	53	11			0.059

## Data Availability

The data used to support the findings of this study are available from the corresponding author upon request.
